# Demonstration of a Novel HIV-1 Restriction Phenotype from a Human T Cell Line

**DOI:** 10.1371/journal.pone.0002796

**Published:** 2008-07-30

**Authors:** Yanxing Han, Xiaojun Wang, Ying Dang, Yong-Hui Zheng

**Affiliations:** Department of Microbiology and Molecular Genetics, Michigan State University, East Lansing, Michigan, United States of America; Institut Pasteur Korea, Republic of Korea

## Abstract

**Background:**

Although retroviruses may invade host cells, a productive infection can be established only after the virus counteracts inhibition from different types of host restriction factors. Fv1, APOBEC3G/F, TRIM5α, ZAP, and CD317 inhibit the replication of different retroviruses by interfering with viral uncoating, reverse transcription, nuclear import, RNA stability, and release. In humans, although APOBEC3G/3F and CD317 block HIV-1 replication, their antiviral activities are neutralized by viral proteins Vif and Vpu. So far, no human gene has been found to effectively block wild type HIV-1 replication under natural condition. Thus, identification of such a gene product would be of great medical importance for the development of HIV therapies.

**Method and Findings:**

In this study, we discovered a new type of host restriction against the wild type HIV-1 from a CD4/CXCR4 double-positive human T cell line. We identified a CEM-derived cell line (CEM.NKR) that is highly resistant to productive HIV-1 infection. Viral production was reduced by at least 1000-fold when compared to the other permissive human T cell lines such as H9, A3.01, and CEM-T4. Importantly, this resistance was evident at extremely high multiplicity of infection. Further analyses demonstrated that HIV-1 could finish the first round of replication in CEM.NKR cells, but the released virions were poorly infectious. These virions could enter the target cells, but failed to initiate reverse transcription. Notably, this restriction phenotype was also present in CEM.NKR and 293T heterokaryons.

**Conclusions:**

These results clearly indicate that CEM.NKR cells express a HIV inhibitory gene(s). Further characterization of this novel gene product(s) will reveal a new antiretroviral mechanism that directly inactivates wild type HIV-1.

## Introduction

Mammals have developed intracellular innate immunity that affords them protection from retroviral infection. This immunity is composed of different host antiviral genes, which are also called restriction factors. So far, at least five different types of restriction factors have been identified as important players for this immunity: FV1, ZAP, APOBEC3G/F, TRIM5α, and CD317.

Fv1 is the first-discovered antiretroviral gene that restricts the replication of murine leukemia virus (MuLV) [1]. It is a *gag*-like gene from an endogenous retrovirus on mouse chromosome 4 [2], [3]. It recognizes a single residue (R110/E110) on the incoming MuLV capsid (CA) protein [4] and inhibits nuclear import of the viral preintegration complex (PIC) [5], [6]. Fv1 restriction is saturable with a high multiplicity of viral infection (MOI). In addition, during the functional cloning of antiviral genes from a rat cDNA library, another MuLV inhibitory gene was identified [7]. This gene encodes a CCCH-type zinc finger protein, and is called Zinc-finger antiviral protein (ZAP). ZAP can directly bind to viral RNAs in the cytoplasm and degrade these targets [8]. It also inhibits the replication of several viruses in the *Alphavirus* genus [9].

Although Fv1 is only expressed in mice, an Fv1-like block was found in non-murine cells. When human and simian cells were infected by the N-tropic MuLV or HIV-1, respectively, viral replication was inhibited by a similar post-entry block [10]–[14]. The block is saturable and the same viral CA proteins are targeted. This gene was identified as Trim5α, which belongs to the tripartite motif (TRIM) family and blocks viral uncoating [15].

During the study of *vif*-deficient HIV-1 replication, a different type of restriction was identified in so-called non-permissive cells [16]. This restriction targets viral reverse transcription and APOBEC3G was first identified to be responsible for this inhibition [17]. APOBEC3G belongs to a small group of proteins in the cytidine deaminase family, which is also known as APOBEC family [18]. This group of proteins includes APOBEC3A, APOBEC3B, APOBEC3C, APOBEC3DE, APOBEC3F, APOBEC3G, and APOBEC3H. The replication of HIV-1 can be inhibited by APOBEC3B, APOBEC3DE, APOBEC3F, APOBEC3G, and A3POBEC3H [17], [19]–[25]. APOBEC3G shows the most powerful anti-HIV-1 activity [17]. Nevertheless, HIV-1 is able to elude this defense mechanism and cause disease in humans for two reasons. First, A3B and A3H are poorly expressed in vivo [20], [22], [26], [27]. Second, HIV-1 produces Vif, which binds to and mediates the destruction of A3DE, A3F, and A3G in 26S proteasomes via recruitment of the Cullin5 ubiquitin E3 ligase [28], [29]. Similarly, the viral protein, Vpu, was found to counter another host restriction for HIV-1 in certain human cell lines that prevent virus from release [30], [31]. This restriction gene was identified as the cell surface protein CD317, which is an interferon α-inducible gene named Tetherin [32]. Thus, no human gene has been identified that effectively blocks the wild type HIV-1 replication under the physiological condition.

CEM.NKR is a natural subclone of the human T lymphoblastoid cell line CEM. Like other cancer cell lines, CEM cells are very sensitive to the natural killer (NK) cell-mediated lysis. However, CEM.NKR was cloned from a subset of CEM cells that survived from this killing [33]. Since CEM.NKR cells showed very low affinity to NK cells, it was speculated that it lost an unknown cell surface antigen(s) for NK cell recognition [33]. Later, it was found that CEM.NKR does not express calnexin, a type I transmembrane protein identified as a major calcium binding protein of the mammalian ER [34]. Calnexin binds to N-linked glycosylated proteins and has a role in the retention of misfolded glycoproteins in the ER [35]. However, genomic knock-in of a functional calnexin gene into CEM.NKR did not increase NK cell sensitivity [36]. Previously, an interaction between calnexin and the uncleaved HIV-1 gp160 glycoprotein was detected [37]. Although it was speculated that calnexin might function as a gp160 chaperone, no functional evidence was obtained [38], [39].

Here, we report that although CEM.NKR expresses normal levels of CD4 and CXCR4, it is highly resistant to a productive HIV-1 replication. Further analysis indicated that this resistance is not due to the lack of cellular factor such as calnexin, but the presence of an unknown inhibitor(s).

## Results

### CEM.NKR is resistant to productive HIV-1 infection

**Figure 1:**
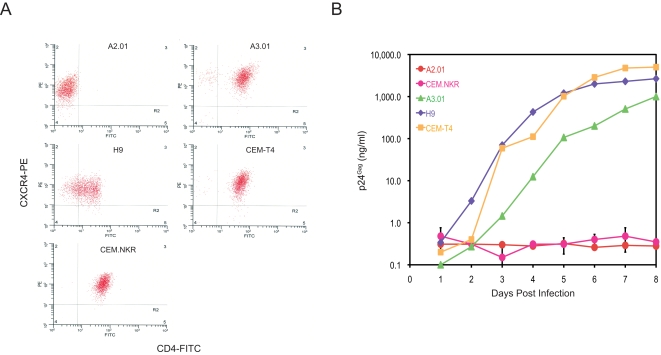
CEM.NKR is resistant to productive infection by HIV-1. A) CEM.NKR cell surface CD4 and CXCR4 expression determined by flow cytometry and compared with the other indicated cell lines. B) HIV-1 infection kinetics. A total of 5×10^5^ of the indicated cells was infected with 100 ng HIV-1 (NL 4-3) and viral growth curves were determined by measuring p24^Gag^ in the supernatant. Results shown are one of three independent experiments. The error bars for CEM.NKR represent standard deviation in these three independent experiments.

Human cell lines that express CD4 and CXCR4 generally should support productive T cell-tropic HIV-1 infection. To discover novel HIV restriction factors, five human T cell lines were chosen for HIV-1 infection experiments. A2.01, A3.01, CEM-T4, and CEM.NKR are originally derived from CEM cells, and H9 is originally from the Hut78 cell line. In addition, A2.01 is an A3.01 variant that has lost CD4 expression. FACS analysis revealed that A3.01, CEM-T4, CEM-NKR, and H9 all expressed comparable levels of CD4 and CXCR4, and A2.01 only expressed CXCR4 (Fig. 1A). Thus, it was assumed that CEM.NKR cells would support a productive HIV-1 infection.

When these T cell lines were inoculated with T cell tropic HIV-1, a robust viral replication was detected in A3.01, CEM-T4, and H9 cells and no viral production was detected in A.201, which is consist with viral receptor expression (Fig. 1B). Surprisingly, a very marginal viral production was detected in CEM.NKR cells. After eight days, the p24^Gag^ levels from CEM.NKR cell supernatant were only 0.35 ng/ml, which was 2,860, 7,600, or 14,342-fold lower than those from A3.01, H9, or CEM-T4 cells. We have tried different T-tropic strains including NL and IIIB and obtained similar results (not shown). Thus, we concluded that CEM.NKR is resistant to productive infection.

**Figure 2:**
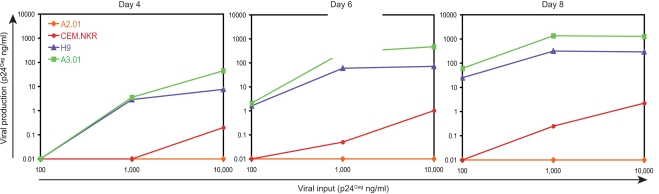
The resistance factor in CEM.NKR is not saturable with HIV-1 virus. Cells were infected with increasing amounts of HIV-1 followed by determination of supernatant p24^Gag^ using ELISA at four, six, and eight days.

To understand whether CEM.NKR cells express a Trim5α-like inhibitory gene, cells were infected with increasing amounts of HIV-1 to see whether this inhibition was saturable. A total of 1×10^5^ cells were inoculated with 100, 1000, or 10,000 ng of HIV-1, which was equivalent to 50, 500, or 5,000 MOI, respectively, and viral replication was observed for 8 days. As presented in Fig. 2, we observed a similar variation in viral production among four different cell lines on day 4, 6, and 8 post-infection. A2.01 cells were resistant to HIV-1 infection even at the highest MOI and viral productions from A3.01 and H9 cells reached the maximal levels at MOI 500 on day 8. In the case of CEM.NKR, viral production was only increased marginally under these conditions. For example, at the highest MOI, it was only around 2 ng/ml on day 8. This level of production was at least 100 and 500-fold lower than those from H9 and A3.01 cells. These results indicate that the resistance of CEM.NKR to HIV-1 was not saturable, which is distinguishable from Trim5α restriction.

### Stepwise analysis of HIV-1 life cycle in CEM.NKR cells

**Figure 3:**
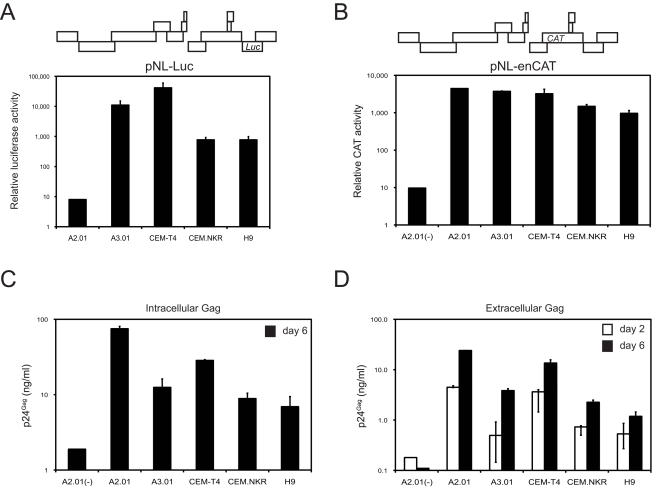
Single-round HIV-1 replication in human T cell lines. A) Expression of viral protein from multiply spliced RNA species. The simple reference diagram at the top of the figure indicates the position of the reporter gene in the viral genome. The indicated cell lines were infected with pNL-Luc reporter viruses. Twenty-four hours later, intracellular luciferase activity was measured. B) Expression of viral protein from singly spliced RNA species. The indicated cell lines were infected with VSV-G pseudotyped pNL-enCAT reporter viruses followed by intracellular CAT activity assay 24 hours later. A2.01(-) served as a negative control where A2.01 cells were infected by the same virus without VSV-G pseudotying. C) Expression of viral protein from un-spliced RNA species. The indicated cell lines were infected by *env*-deficient HIV-1 pseudotyed with VSV-G. Six days later the levels of intracellular Gag protein were determined by ELISA. A2.01(-) served as a negative control where A2.01 cells were infected by the same virus without VSV-G pseudotying. D) Levels of viral release. The infection experiment was performed as in C) and levels of viral release were determined by p24^Gag^ ELISA. Error bars in these experiments represent standard deviations in three independent experiments.

The resistance of CEM.NKR cells led us to investigate which step in the viral life cycle was disrupted. Initially, we determined whether HIV-1 could enter CEM.NKR cells and express viral proteins. We used two previously described HIV-1 reporter viruses: pNL-Luc and pNL-enCAT [40]. pNL-Luc contains a firefly luciferase (Luc) gene in the Nef open reading frame (ORF) and the expression of luciferase is from the 2 kb viral transcripts (Fig. 3A); pNL-enCAT contains a chloramphenicol acetyl transferase (CAT) gene in the gp120 ORF and the expression of CAT is from the 4 kb viral transcripts (Fig. 3B). Both Luc and CAT-reporter viruses were produced by transfection of 293T cells; the CAT-reporter virus was pseudotyped with the vesicular stomatitis virus envelope glycoprotein (VSV-G) to provide a functional envelope. When cells were infected with the Luc-reporter viruses, robust Luc activities were detected in A3.01, CEM-T4, CEM.NKR, and H9 cells but not A2.01 lacking viral receptors (Fig. 3A). This result demonstrated that HIV-1 could efficiently enter CEM.NKR cells, and also make the early gene products. Consistently, when cells were infected with the CAT-reporter viruses, robust CAT activities were detected in A2.01, A3.01, CEM-T4, CEM.NKR, and H9 cells but not in A2.01(-), where A2.01 cells were infected by the same virus without VSV-G pseudotyping (Fig. 3B). This result demonstrated that HIV-1 could make the 4 kb transcripts and produce related proteins in CEM.NKR cells as well as in A3.01, CEM-T4, H9, and A2.01 cells.

Next, we determined whether HIV-1 could make the 9 kb transcripts where the Gag proteins were translated. Cells were infected with *env*-deficient HIV-1 pseudotyped with VSV-G and lysed 6 days post-infection to measure newly synthesized Gag proteins. As presented in Fig. 3C, comparable levels of Gag proteins were detected in A2.01, A3.01, CEM-T4, CEM.NKR, and H9 cells, which were significantly higher than those in A2.01 cells infected by the same virus without VSV-G pseudotyping. This result demonstrated that HIV-1 could make Gag proteins in CEM.NKR cells, suggesting that the 9 kb viral transcripts are normally produced.

Finally, we determined whether viral particles were released from these cells. As in Fig. 3C, culture supernatants were collected 2 or 6 days post-infection and levels of extracellular Gag protein were determined by ELISA (Fig. 3D). The Gag protein levels from CEM.NKR cell culture increased 3-fold, whereas a 5, 7, 3, or 2-fold increase was detected from A2.01, A3.01, CEM-T4, or H9 cell culture, respectively. No increase was detected from A2.01 cell culture inoculated with the same virus without VSV-G pseudotyping, and their values were significantly lower than those samples infected by VSV-G pseudotyped viruses. This result demonstrated that HIV-1 particles are assembled and released from CEM.NKR cells. Thus, there is no apparent defect in HIV-1 replication during the first cycle in CEM.NKR cells.

### Characterization of CEM.NKR released HIV-1

**Figure 4:**
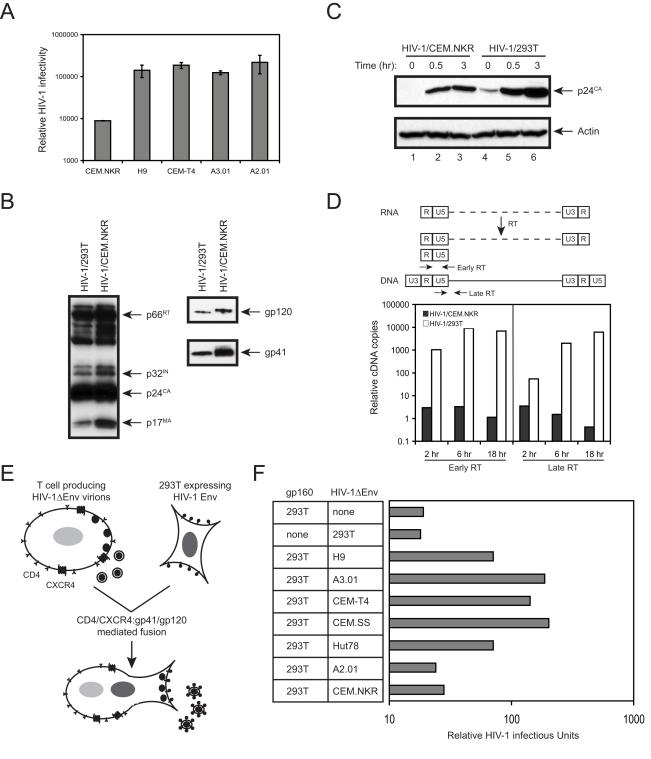
Characterization of CEM.NKR-released virions. A) Viral infectivity assay. The indicated T cells were infected with HIV-1 pseudotyped by VSV-G and equal amounts of viruses as measured by p24^Gag^ were collected to infect TZM-bl cells. Viral infectivity was determined by measuring cellular luciferase activity twenty-four hours' later. B) Viral protein expressions. Proteins from virions from HIV-infected CEM.NKR cells or transfected 293T cells were analyzed by Western blotting using human anti-HIV polyclonal, mouse anti-gp41 monoclonal, and mouse anti-gp120 monoclonal antibodies. C) Viral entry assay. Viruses purified in B) were incubated with TZM-bl cells for 0, 0.5, and 3 hours followed by Western blotting analysis of intracellular viral p24^Gag^. D) Real-time PCR analysis of viral reverse transcripts. Viruses purified in B) were used to infect CEM-SS cells and cellular DNAs were extracted at the indicated time points. Levels of the early (left panel) or late reverse transcripts (right panel) were determined by real-time PCR. The position of the early and late primer pairs is indicated by arrows above the panels. E) A schematic description of the transient trans-complementation assay via heterokaryon formation. T cells were infected with *env*-defective HIV-1 virus pseudotyped with VSV-G and 293T cells transfected with Env-expressing vector pNLΔGag. Cells were then co-cultured for heterokaryon formation and infectious particles were detected by infection of TZM-bI cells. F) Infectivity of HIV-1 produced from heterokaryons. The Env-expressing 293T cells were co-cultured with indicated T cells producing *env*-defective HIV-1. After 48 hours, viral infectivity from the co-culture was determined in TZM-bl cells. Results shown here were from one of three independent experiments.

To know whether a block might occur during the next round of replication, we measured viral infectivity. To increase viral production from CEM.NKR cells, viruses were pseudotyped with VSV-G before infection. VSV-G pseudotyped HIV-1 viruses were first created from 293T cells and used to infect CEM.NKR, H9, CEM-T4, A3.01, and A2.01 cells. Viruses were collected from these cell cultures 24 hours post-infection. After normalized by p24^Gag^, the same amounts of viruses were collected to infect TZM-bl cells, a HIV-reporter cell line expressing the Luc gene under HIV LTR control. As presented in Fig. 4A, the infectivity of virions produced from CEM.NKR cells was at least 15-fold lower than that from H9, CEM-T4, A3.01, and A2.01 cells. It should be noted that this decrease was underestimated because TZM-bI cells produce certain levels of luciferase activity even without viral infection. Thus, CEM.NKR released virions are poorly infectious, which explains why productive viral infection could not be established.

To understand why virions are not infectious, we determined whether these virions lost any of important viral components. To prepare high quantity purified virions, CEM.NKR cells were infected with HIV-1 carrying a neomycin-resistant marker and a persistently infected cell line was created by G418 selection. Virions were then purified from culture supernatant by ultracentrifugation and viral proteins were determined by Western blotting. As a control, virions were also produced from 293T cells. As presented in Fig. 4B, viral reverse transcriptase (p66^RT^), integrase (p32^IN^), capsid (p24^CA^), matrix (p17^MA^), gp120, and gp41 proteins were all detected in virions from CEM.NKR cells, indicating that viral Gag, Pol, and Env proteins were properly expressed, processed, assembled, and incorporated into virions. The migration of gp120 proteins from CEM.NKR cells in SDS gel was slightly slower than that from 293T cells, which might reflect a difference in protein glycosylation in these cells.

Next, we determined virion cell entry. Virions from CEM.NKR and 293T cells were incubated with TZM-bl cells at 37 °C. After 0, 0.5, and 3 hours, cells were washed extensively and trypsinized to remove viruses that were only attached to the cell surface. Cells were then lysed and levels of intracellular p24^CA^ were determined by Western blotting. A time-dependent increase of intracellular p24^CA^ proteins was observed in cells infected with both viruses, but the levels of increase in cells infected with 293T-derived viruses were higher than those infected with CEM.NKR-derived viruses (Fig. 4C). Thus, although we could conclude that virions from CEM.NKR cells were able to enter the target cell, a minor defect in viral entry could not be completely excluded.

Finally, we determined whether viruses could initiate reverse transcription in the target cells. CEM-SS cells were infected with the same amounts of viruses produced from either CEM.NKR or 293T cells. After 2, 6, or 18 hours of infection, cellular DNAs were extracted and viral early or late reverse transcription (RT) products were quantitated by Real-time PCR (Fig. 4D). The levels of both early and late RT products in cells infected with virions from CEM.NKR were extremely low and decreased in a time-dependent manner. In contrast, levels of both viral RT products in cells infected with virions from 293T were very high and increased in a time-dependent manner. The differences in the levels of viral RT products in cells infected with these two different viruses could reach 10^3^ to 10^4^-fold after 18 hours of infection. Since such huge differences could not be simply explained by the difference in entry between these two viruses, we concluded that virions from CEM.NKR cells fail to initiate reverse transcription, which becomes a major post-entry block to the virus in the target cells.

### CEM.NKR cells express a HIV-1 inhibitor(s)

We proposed two hypotheses to explain why viruses from CEM.NKR cells are not infectious: 1) CEM.NKR cells lack cofactor(s) essential for HIV-1 replication; and 2) CEM.NKR cells expresses dominant inhibitor(s) that blocks HIV-1 replication. A trans-complementation assay that detects infectious viral particles from heterokaryons was used to test these two hypotheses [41], [42]. Heterokaryons were formed by HIV-1 Env and CD4/CXCR4 mediated cell fusion of non-permissive CEM.NKR cells and permissive 293T cells. Infectious viral particles were detected by infection of TZM-bI cells. If viruses produced from heterokarons are infectious, CEM.NKR cells should lack cofactor(s); otherwise, CEM.NKR cells should express inhibitor(s). To ensure that particles are produced exclusively from heterokaryons, viral proteins were expressed separately from these two cell types. In addition, since CEM.NKR lacks calnexin that might have unknown function for viral gp160, we chose to express gp160 in 293T cells and the *env*-deficient viral particles were produced from CEM.NKR (Fig. 4E).

Initial control experiments were performed to make sure this experiment worked as desired. As presented in Fig. 4F, no infectious particles were recovered from 293T cells expressing either Env protein or producing *env*-deficient viral particles; or from co-cultures between 293T and A2.01 cells. In sharp contrast, when A2.01 cells were replaced with H9, A3.01, CEM-T4, CEM.SS, and Hut78 cells, high titers of infectious particles could be recovered from the co-culture, indicating successful heterokaryon formations between 293T and these T cells. Notably, when Env-expressing 293T cells were co-cultured with CEM.NKR cells producing *env*-deficient viruses, no infectious particles were detected. It is unlikely that CEM.NKR failed to fuse with 293T cells because CEM.NKR cells express high levels of CD4 and CXCR4 (Fig. 1A), which is functional for viral entry (Fig. 4C). Thus, the only possibility is that CEM.NKR cells express an inhibitor(s) that potently block HIV-1 replication. This result also excludes the responsibility of calnexin for HIV restriction in CEM.NKR cells.

### CEM.NKR-CCR5 and CEM.NKR-CCR5-Luc cell lines are also resistant to productive HIV-1 infection

**Figure 5:**
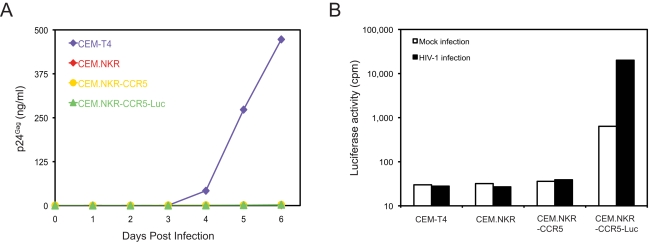
HIV resistance in two other CEM.NKR-derived cell lines. A) HIV-1 infection kinetics. A total of 5×10^5^ CEM-T4, CEM.NKR, CEM.NKR-CCR5, or CEM.NKR-CCR5-Luc cells was infected with 100 ng HIV-1 (NL 4-3) and viral infection was determined by measuring p24^Gag^ in the supernatant over 6 days. B) Cellular luciferase activity. These cells were infected with HIV-1 as in A). Three days later, cells were lysed and cellular luciferase activity was determined.

Two additional cell lines have been generated from CEM.NKR. The first is CEM.NKR-CCR5 that was created by stable transduction of CEM.NKR with a CCR5-expressing vector [43]. The second is CEM.NKR-CCR5-Luc that was created by stable transfection of CEM.NKR-CCR5 cells with a HIV-2 LTR-Luc expression vector [44]. These two cell lines were previously shown to express similar levels of CD4 and CXCR4 levels when compared to the parent cell line CEM.NKR [44]. To test whether these two cell lines retained the HIV-resistance phenotype, they were infected with HIV-1 strain NL4-3. As shown in Fig. 5A, CEM.NKR, CEM.NKR-CCR5, and CEM.NKR-CCR5-Luc were equally resistant to productive HIV-1 infection, whereas the CEM-T4 cell line was highly permissive for HIV-1 infection.

To determine whether HIV-1 could enter CEM.NKR cells, we measured intracellular luciferase activity in CEM.NKR-CCR5-Luc cells. Before infection we found only a background level of luciferase activity at around 1,000 cpm. This activity increased at least 30-fold upon viral infection. Cells without the luciferase gene (CEM-T4, CEM.NKR, and CEM.NKR-CCR5) were negative (Fig. 5B). Thus, we conclude that HIV-1 can enter CEM.NKR cells. Together, these results are consistent with the conclusion that HIV-1 resistance in CEM.NKR cells is due to a post-entry block during the 2^nd^ round of viral infection.

## Discussion

We have demonstrated that CEM-NKR cells express a novel restriction factor(s) that poses a major post-entry block to HIV-1. Three pieces of evidence indicate that this restriction might not be caused by an entry block due to the lack of calnexin. First, the fact that gp120 and gp41 were both detected from virions released from CEM.NKR (Fig. 4B) confirms that gp160 is folded and processed correctly in the ER. Second, virus could enter the target cell (Fig. 4C). Third, the trans-complementation of functional gp160 proteins from 293T cells did not release virus from this block (Fig. 4F). However, it should be noted that our data cannot completely rule out a possible entry block that may play a minor role in this restriction. In addition, although APOBEC3B and APOBEC3H can inactivate wild type HIV-1, we failed to detect their mRNAs from CEM.NKR (data not shown). Moreover, since this inhibition is not saturable (Fig. 2), it does not have the properties of a Trim5α-like block. Nevertheless, we cannot completely exclude the antiviral activity from the other Trim family members in CEM.NKR cells [45].

Initially, we were very surprised by that CEM.NKR cells were highly resistant to HIV-1 replication because two CEM.NKR-derived cell lines, CEM.NKR-CCR5 and CEM.NKR-CCR5-Luc, were developed to study HIV-1 infection [43], [44]. These cell lines are available through NIH AIDS Research and Reference Reagent Program. Our results confirmed that HIV-1 can enter these cells, but they are equally resistant to productive HIV-1 infection. Interestingly, although CEM.NKR-CCR5 is recommended for infection with primary HIV isolates and neutralization assays, it is indeed acknowledged in the data sheet that this cell line does not secrete infectious virus. Moreover, it is not surprising that CEM.NKR-CCR5-Luc can be used as HIV-indicator cell because we also showed that CEM.NKR is infectable with HIV-1. Although this cell line was reported to be permissive for viral replication, the original report shows a peak of viral production of only about 10 ng/ml [44]. This value is just slight higher than that we detected in Fig. 2. Although CEM-NKR cells can be infected with HIV-1, they do not effectively produce infectious particles. Taken together, these results have permitted the identification of a new type of HIV-1 restriction factor(s) in CEM.NKR cells. Further elucidation of the nature of this restriction factor will uncover a novel antiretroviral mechanism that effectively inhibits wild type HIV-1 replication.

## Materials and Methods

### Plasmids, cell lines, and viruses

HIV-1 proviral constructs pNL4-3, pNL-Luc, and pNL-enCAT were described before [25], [40]. pNL-Neo was created by replacing the firefly luciferase in pNL-Luc with the neomycin-resistant gene by NotI/XhoI double digestion. pNLΔGag and pNLΔEnv were created by SphI/AgeI double or NheI single digestion of pNL4-3 followed by large Klenow fragment treatment before T4 ligation. The NheI site is still active in pNLΔEnv although the *env* gene was inactivated by a frame-shift.

The HIV indicator cell line TZM-bI and human T cell lines HUT 78, H9, PM1, CEM-SS, CEM.NKR, CEM.NKR-CCR5, CEM.NKR-CCR5-Luc, A3.01, and A2.01 were from NIH AIDS Research and Reference Reagent Program. T cells were cultured in RPMI 1640 with 10% fetal bovine serum (HyClone). 293T and TZM-bI were cultured in DMEM with 10% bovine calf serum (HyClone).

Viruses were produced from 293T cells by the standard calcium phosphate transfection of the proviral constructs. HIV-1 IIIB were obtained from NIH AIDS Research and Reference Reagent Program and propagated in H9 cells.

### Antibodies

The following antibodies were obtained through the AIDS Research and Reference Reagent Program: human anti-HIV immunoglobulin (#3957) from NABI, HIV-1 IIIB gp41 hybridoma (#526) from George Lewis, HIV-1 IIIB gp120 hybridoma (#902) from Bruce Chesebro, HIV-1 p24 hybridoma from Bruce Chesebro and Hardy Chen. Other antibodies used included a polyclonal rabbit anti-actin antibody (C-11) (Santa Cruz Biotechnology), PE-conjugated mouse anti-human CXCR4 and FITC-conjugated mouse anti-human CD4 (BD Biosciences), and HRP-conjugated anti-rabbit, human, or mouse IgG secondary antibodies (Pierce). Detection of the HRP-conjugated antibody was performed using Supersignal Wetpico Chemiluminescence Substrate kit (PIERCE).

### HIV-1 infection of human T cell lines

1×10^5^ cells were incubated with 100 ng wild type HIV viruses at 37°C for three hours. After removal of the inocula followed by three extensive washings, cells were cultured in 24-well plates for eight days. Culture supernatants were then collected daily for measurement of p24^Gag^ by ELISA.

### Real-time PCR measurement of viral reverse transcripts

5×10^6^ CEM-SS cells were infected with HIV-1 equivalent to 200 ng p24^CA^ and cellular DNAs were extracted 2, 6, and 18 hours after infection using the DNeasy kit (QIAGEN). After digestion with DpnI, viral reverse transcripts were determined by real-time PCR using TaqMan® Gene Expression Master Mix kit (Applied Biosystems). The early reverse transcripts (strong stop) were amplified by previously described primers oHC64 and oHC65 and quantitated by a fluorescence labeled probe oHC66 [46]. The late reverse transcripts were amplified by previously described primers MH531 and MH532 and the labeled probe was LRT-P [47]. Reactions were analyzed by the ABI 7900HT (Applied Biosystems).

### CAT assay

Chloramphenicol acetyl transferase (CAT) activity was determined as before [48]. Briefly, cells were lysed in 0.25 M Tris-HCl at pH 7.5 containing 0.1% Triton X-100. Lysates were incubated at 65 °C for 5 min to inactivate the endogenous CAT enzyme. Nuclei were then removed from the lysates by centrifugation. Cleared lysates (100 µl) were mixed with CAT reaction solution (50 µl) containing 0.5 µl acetyl-conenzyme A (4.90 Ci mmol^−1^), 41.5 µg chloramphenicol and 5 µl 0.25M Tris-HCl at pH 7.5. Finally, 3 ml Econoflour (Packard Bioscience, Netherland) was added to the reaction before scintillation counting. Activity was expressed as the slope of the enzyme activity.

### Heterokaryon formation

A previously established protocol was adopted [41], [42]. Briefly, 293T cells were seeded in six-well plates at 8×10^5^/well in 2 ml medium. Twelve hours later, cells were transfected with 6 µg of HIV Env expression vector pNLΔGag and washed with PBS four hours' later. Simultaneously, 8×10^5^ T cells were infected with 500 ng of VSV-pseudotyped Env-defective HIV-1 from pNLΔEnv-transfected 293T cells at 37°C for three hours. After removal of the inocula and extensive washing, infected T cells were added to the Env-expressing 293T cell culture. After 48 hours, supernatants from these co-cultures were collected to infect TZM-bI cells. Viral infectivity was finally determined by measuring cellular luciferase activities after another 48 hours.
